# TRIM7 suppresses transmissible gastroenteritis virus replication by targeting the degradation of N protein and activating RIG-I-mediated type I IFN antiviral response

**DOI:** 10.1186/s13567-025-01610-z

**Published:** 2025-11-05

**Authors:** Weiyun Qin, Yunxiao Xie, Haijiang Liang, Zuolin Wu, Songbai Yang, Xiaolong Zhou, Ayong Zhao

**Affiliations:** 1 Key Laboratory of Applied Technology On Green-Eco-Healthy Animal Husbandry of Zhejiang Province, College of Animal Science and Technology, College of Veterinary Medicine, Zhejiang A&F University, Hangzhou, 310000 China; 2 College of Animal Science and Technology, Yangzhou University, Yangzhou, 225009 China

**Keywords:** Transmissible gastroenteritis virus, TRIM7, ubiquitination, RIG-I signaling pathway, type I interferon

## Abstract

Transmissible gastroenteritis virus (TGEV) is a major enteric pathogen causing severe diarrhea in swine, with substantial implications for the biosecurity of intensive pig production worldwide. The E3 ubiquitin ligase TRIM7 has emerged as a key regulator of host antiviral immunity, yet its role in TGEV infection remains elusive. Here, we report that TRIM7 is abundantly expressed in porcine intestinal tissue and significantly upregulated upon TGEV challenge in IPEC-J2 cells. Functional studies demonstrated that TRIM7 restricts TGEV replication in IPEC-J2 cells through two distinct mechanisms. Firstly, molecular interaction and co-immunoprecipitation assays revealed that TRIM7 mediates ubiquitin-dependent degradation of the TGEV nucleocapsid (N) protein. Secondly, RNA-seq analysis combined with RIG-I signaling modulation experiments established that TRIM7 enhances RIG-I-mediated type I interferon (IFN) signaling to suppress viral propagation. These findings uncover a dual antiviral role for TRIM7 in countering TGEV infection, highlighting its potential as a therapeutic target for TGEV control.

**Supplementary Information:**

The online version contains supplementary material available at 10.1186/s13567-025-01610-z.

## Introduction

Transmissible gastroenteritis (TGE) is an acute and highly contagious enteric disease affecting pigs, caused by the transmissible gastroenteritis virus (TGEV), a pathogen of significant concern in the livestock industry. TGEV belongs to the genus Alphacoronavirus within the family *Coronaviridae* and is the etiological agent responsible for severe viral enteritis in pigs. The infection primarily targets the small intestine, resulting in extensive epithelial damage, compromised mucosal immunity, and increased expression of inflammatory cytokines. These factors may contribute to chronic inflammation and persistent viral shedding [1]. The severity of TGEV-induced disease is inversely correlated with the age of infected piglets. Clinical symptoms such as vomiting, diarrhea, and dehydration appear within two weeks post-infection. Notably, the mortality rate in piglets under two weeks of age can exceed 80%, which poses a significant threat to both swine health and production [2–5]. TGEV is primarily transmitted through the fecal–oral route, but respiratory and lactogenic transmission also contribute to the spread of the virus [6]. Co-infections with other viral or bacterial pathogens frequently exacerbate clinical symptoms, thereby complicating disease management. Since its initial identification in Taiwan, China, in 1958, extensive research has highlighted the high pathogenicity and infectivity of TGEV [7]. Despite advancements in disease control, TGE remains a major threat to China's swine industry, particularly during the winter and early spring, leading to substantial economic losses [8]. Additionally, TGEV continues to evolve and undergo genetic recombination, raising concerns about vaccine efficacy and the potential for cross-species transmission [6]. Therefore, elucidating the molecular mechanisms underlying TGEV replication and identifying novel host antiviral targets could provide valuable insights for developing effective strategies for disease prevention and control.

Tripartite motif-containing proteins (TRIMs) are a family of E3 ligases that play crucial roles in various cellular functions, including the regulation of innate immunity and the antiviral response. TRIMs are known to orchestrate the ubiquitination of target proteins, regulate proteolytic degradation, assemble signaling complexes, modulate subcellular localization, and influence the dynamics of the host transcriptome and proteome [9–13]. As an E3 ubiquitin ligase, TRIM7 plays a pivotal role in the production of interferon (IFN), signaling cascades, and the activation of NF-κB, thereby contributing to various aspects of antiviral immunity. These aspects include the recognition of pathogen-associated molecular patterns (PAMPs) mediated by pattern recognition receptors (PRRs), the regulation of transcription factors, the facilitation of signal transduction complex assembly, and the degradation of inhibitory molecules [10, 13]. There is increasing evidence supporting TRIM7 as a crucial regulator of host immune responses. It plays a pivotal role in enhancing viral resistance and fine-tuning immune signaling pathways [14–16]. TRIM7 exerts its immunoregulatory effects by catalyzing the ubiquitination of various substrates, including adaptor proteins (e.g., MAVS and STING) and transcription factors (e.g., NF-κB and IRF3) [17], this process results in both positive and negative regulatory influences on immune pathways.

However, viruses have evolved sophisticated immune evasion strategies to counteract TRIM7-mediated antiviral defenses. Certain viruses can target TRIM7 for degradation or sequester it from its substrates, effectively suppressing its function and facilitating viral persistence [18]. Given TRIM7’s crucial role in host immunity, understanding the interaction between TRIM7 and antiviral defense mechanisms is vital for developing novel antiviral therapeutics. Although TRIM7 has been implicated in antiviral responses against certain human enteroviruses, its specific role in TGEV infection remains largely unexplored and requires further investigation.

## Materials and methods

### Tissues, cell lines, virus, and plasmids

Twelve porcine tissue cDNA samples were cryopreserved in our laboratory. The IPEC-J2, HEK 293 T, and PK15 cell lines were obtained from the American Type Culture Collection (ATCC) and were cultured in Dulbecco’s Modified Eagle’s Medium (DMEM) (Gibco, USA), supplemented with 10% fetal bovine serum (FBS). The TGEV (SHXB) strain was also stored in our laboratory.

### Cell transfection

Small interfering RNA (siRNA) oligos targeting TRIM7 were synthesized by Shanghai GenePharma Co., Ltd. (Shanghai, China). Detailed oligonucleotide sequences are listed in Table 1. The pCDNA3.1 ( +) vector was utilized to construct TRIM7 overexpression plasmids. All transfections were performed using jetPRIME (Polyplus, No. 101000046) in accordance with the manufacturer’s protocol. Briefly, 20 μM siRNA or 2 μg of plasmid DNA (for a 6-well plate) was diluted in 200 μL of jetPRIME buffer and mixed thoroughly by pipetting. Subsequently, 4 μL of jetPRIME reagent was added, followed by vortexing for 10 s. The mixture was incubated at room temperature for 10 min before being added to the culture medium. The plate was gently rocked and then returned to the incubator. At 24 h post-transfection, the cells were infected with TGEV at a multiplicity of infection (MOI) of 0.1 and collected at different hour post-infection (hpi) for subsequent experiments.

**Table 1:** **Details of oligonucleotide sequences**

Name	Sequences (5′ to 3′)
si-*TRIM7*-1	F: GGCACCUAUUUGCGAAUCUTT
	R: AGAUUCGCAAAUAGGUGCCTT
si-*TRIM7*-2	F: CCCUAAGCAAGUGCAGCAATT
	R: UUGCUGCACUUGCUUAGGGTT
si-*TRIM7*-3	F: GCGAACCUCUCAAGCUCUATT
	R: UAGAGCUUGAGAGGUUCGCTT
Negative control	F: UUCUCCGAACGUGUCACGUTT
	R: ACGUGACACGUUCGGAGAATT

### Quantitative real-time PCR (qRT-PCR)

Total RNA was extracted using RNAiso Plus (TaKaRa, No. 9108) and reverse transcribed with the HiScript III 1st Strand cDNA Synthesis Kit (Vazyme, No. R312-01). The qRT-PCR was conducted using the TB Green PCR kit (TaKaRa, No. RR820A). All qRT-PCR primers are listed in Table 2. GAPDH served as the internal reference gene for normalization.

**Table 2:** **The primers of qRT-PCR**

Genes	Primer sequences (5′-3′)	Length (bp)
*GAPDH*	F: TGACCCCTTCATTGACCTCC	160
	R: CCATTTGATGTTGGCGGGAT	
*TRIM7*	F: ATGGCGGCGGTGGGGCCG	235
	R: TCAAGGCCAGATTCGCAA	
*TGEV-N*	F: CAATTCCCGTGGTCGGAAGA	151
	R: TTTACGTTGGCCCTTCACC	
*TGEV-E*	F: GGAAGGACAGTTATTATTGTTCCA	85
	R: GCATACAACCCCGATGGAGC	
*TGEV-M*	F: GTGCATTAGGAAGAAGCTATG	115
	R: TTCATACCACCTGCAATTTTG	
*RIG-I*	F: CAGTGCAATCTGGTCATCCTAT	159
	R: GGAAACACTTGCTCCCTCTT	
*MDA5*	F: GGTGCAAAGCTTCAGAGACC	151
	R: GAGCCTGCACAAACATCCTA	
*IL-6*	F: CTCTGTCTTAGGGCGTCC	164
	R: CAAGGAGGTACTGGCAGAAA	
*ISG15*	F: GGTGCAAAGCTTCAGAGACC	151
	R: GTCAGCCAGACCTCATAGGC	
*IRF7*	F: CCACACCTTTCCACCCCAAA	179
	R: TTGTTGCTTCTCAGTTCTCTTCA	
*NF-κB*	F: CCCATGTAGACAGCACCACCTATG	132
	R: ACAGAGGCTCAAAGTTCTCCACCA	
*IFN-β*	F: GCTAACAAGTGCATCCTCCAAA	124
	R: CCAGGAGCTTCTGACATGCCA	
*IFN-α*	F: GATCGGTCCCCAAAGGGATG R: CCACTTGGTGGTTTGTGATG	147

### Western blot analysis

Total proteins were extracted and quantified using the BCA Protein Assay Kit (Beyotime Biotechnology, No. P0012). 20 μg of protein were separated by 10% SDS-PAGE and transferred onto 0.22 μm PVDF membranes. The membranes were blocked with 5% skim milk and incubated overnight at 4 °C with primary antibodies. After washing, the membranes were incubated with the secondary antibodies, and protein bands were visualized using an ECL detection system (Bio-Rad, USA). HSP90 was used as the loading control, and details of the primary and secondary antibodies are provided in Table 3.

**Table 3:** **Details of antibodies**

Antibodies	Source	Application
TGEV N	Abcam	western blot, IFA
TRIM7	proteintech	western blot
Ubiquitin	Abcam	western blot
HSP90	Abcam	western blot
Flag	Abcam	western blot, Co-IP
HA	Abcam	western blot, Co-IP
IgG	proteintech	Co-IP
DAPI	Beyotime Biotechnology	IFA

### Indirect immunofluorescence assays

Cells were seeded onto 24-well culture slides, fixed with 4% paraformaldehyde, and permeabilized with 0.05% Triton X-100. Following a 2 h blocking step with 5% bovine serum albumin (BSA), the slides were incubated overnight at 4 °C with primary antibodies. After washing, the slides were treated with the corresponding secondary antibodies for 2 h, followed by DAPI nuclear staining. The stained cells were then observed and photographed using a fluorescence microscope.

### TCID_50_ assay

PK15 cells were seeded in 96-well plates and cultured until 90% confluence. Serial ten-fold dilutions of the virus were prepared and added to the cells, followed by a 1-h incubation at 37 °C to allow viral adsorption. After the virus was removed, fresh medium was added to each well, and cells were cultured for 72 h. Cytopathic effects (CPE) were monitored daily. The 50% tissue culture infective dose (TCID_50_) was calculated using the Reed-Muench method based on the presence or absence of CPE.

### Homology modeling

The complete sequences of TRIM7 (A0A287A7M1) and the TGEV N (Q1W2N6) were obtained from UniProt [19]. AlphaFold Server [20] was utilized to generate high-accuracy biomolecular structure predictions for both TRIM7 and the TGEV N protein. The chains of the TRIM7 and the TEGV N protein are labelled as chain A and chain B, respectively. The molecular docking results were visualized using ChimeraX 1.9 [21].

### Co-immunoprecipitation (Co-IP) assay

IPEC-J2 cells were cultured in 75 cm^2^ flasks, harvested, and lysed in Triton X-100 lysis buffer (40 mM Tris, 120 mM NaCl, 1% Triton X-100, 1 mM NaF, 1 mM Na₃VO₄) supplemented with a protease inhibitor cocktail. The co-immunoprecipitation (Co-IP) assay was performed using the Protein A/G Magnetic Beads Kit (MCE, No. HY-K0202) according to the manufacturer’s protocol. Briefly, 50 μL of Protein A/G Magnetic Beads was incubated with the primary antibody for 2 h in PBST (1 × PBS + 0.5% Tween-20, pH 7.4). The bead-antibody complex was then incubated overnight at 4 °C with the cell lysate supernatant. The beads were washed four times with wash buffer, and SDS-PAGE loading buffer was added. The eluted proteins were analyzed by SDS-PAGE, followed by western blotting using ubiquitin, Flag and HA antibodies.

### RNA-seq analysis

RNA-seq was performed on TRIM7-overexpressing and control IPEC-J2 cells, with three biological replicates per group. Briefly, RNA libraries were constructed using 3 μg of total RNA per sample, following the protocols outlined in the NEBNext^®^ Ultra^™^ II RNA Library Prep Kit for Illumina^®^ (NEB, No. E7770S). Fragment purification was performed using the AMPure XP system (Beckman Coulter, USA), selecting fragments ranging from 150 to 200 bp. cDNA digestion was carried out with USER Enzyme, followed by PCR amplification. The final purified PCR products were analyzed using the Agilent Bioanalyzer 2100 system (Agilent Technologies, USA) and the TruSeq PE Cluster Kit v3-cBot-HS (Illumina, USA). Differentially expressed genes (DEGs) were identified based on a *p*-value < 0.05.

### Statistical analysis

All statistical analyses were performed using SPSS Statistics 26. A two-sided Student’s *t*-test was employed for comparisons between two groups, while one-way analysis of variance (ANOVA) was utilized for comparisons involving multiple groups. Statistical significance was set at *P* < 0.05, and the results were presented as follows: ns: not significant; *P* < 0.05 (*); *P* < 0.01 (**); *P* < 0.001 (***).

## Results

### TGEV infection induces an increase in TRIM7 expression

**Figure 1:**
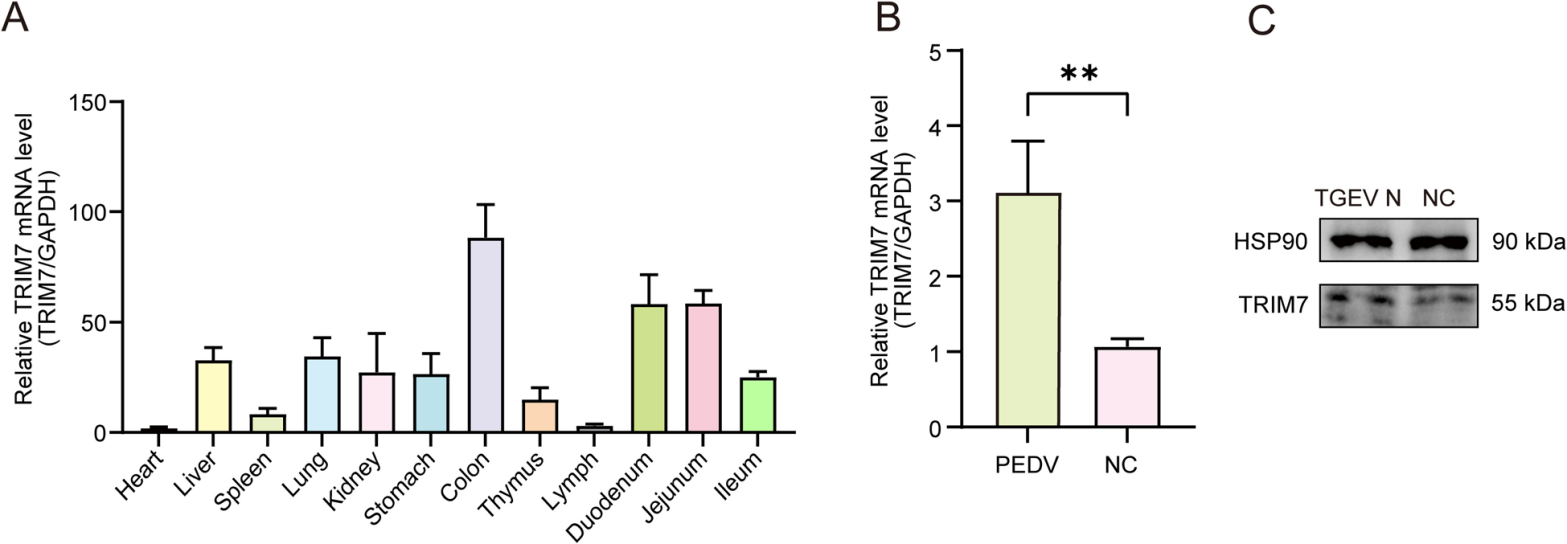
**Tissue expression profile of TRIM7 and its differential expression following TGEV infection. A** TRIM7 expression in 12 porcine tissues, normalized to heart expression. **B** mRNA expression levels of TRIM7 in IPEC-J2 cells 24 h post-TGEV infection. **C** Protein expression levels of TRIM7 in IPEC-J2 cells 24 h post-TGEV infection. *P < 0.01.

In this study, we initially examined the tissue expression profile of TRIM7 in pigs. qRT-PCR results revealed that TRIM7 was highly expressed in the colon, duodenum, and jejunum, while its expression was relatively low in the heart and lymph nodes (Figure 1A). Given that the gastrointestinal tract serves as the first line of defense against TGEV infection, the elevated expression of TRIM7 in these tissues suggests that it may play a role in resisting TGEV infection. Subsequently, we used qRT-PCR to assess TRIM7 mRNA expression in IPEC-J2 cells infected with TGEV for 24 h. The results showed a significant upregulation of TRIM7 expression (*P* < 0.01) (Figure 1B), which was further validated at the protein level through western blot analysis (Figure 1C). These findings indicate a strong correlation between TRIM7 expression and TGEV infection, warranting further investigation into the regulatory relationship between TRIM7 and TGEV.

### TRIM7 is a potential host factor against TGEV infection

**Figure 2:**
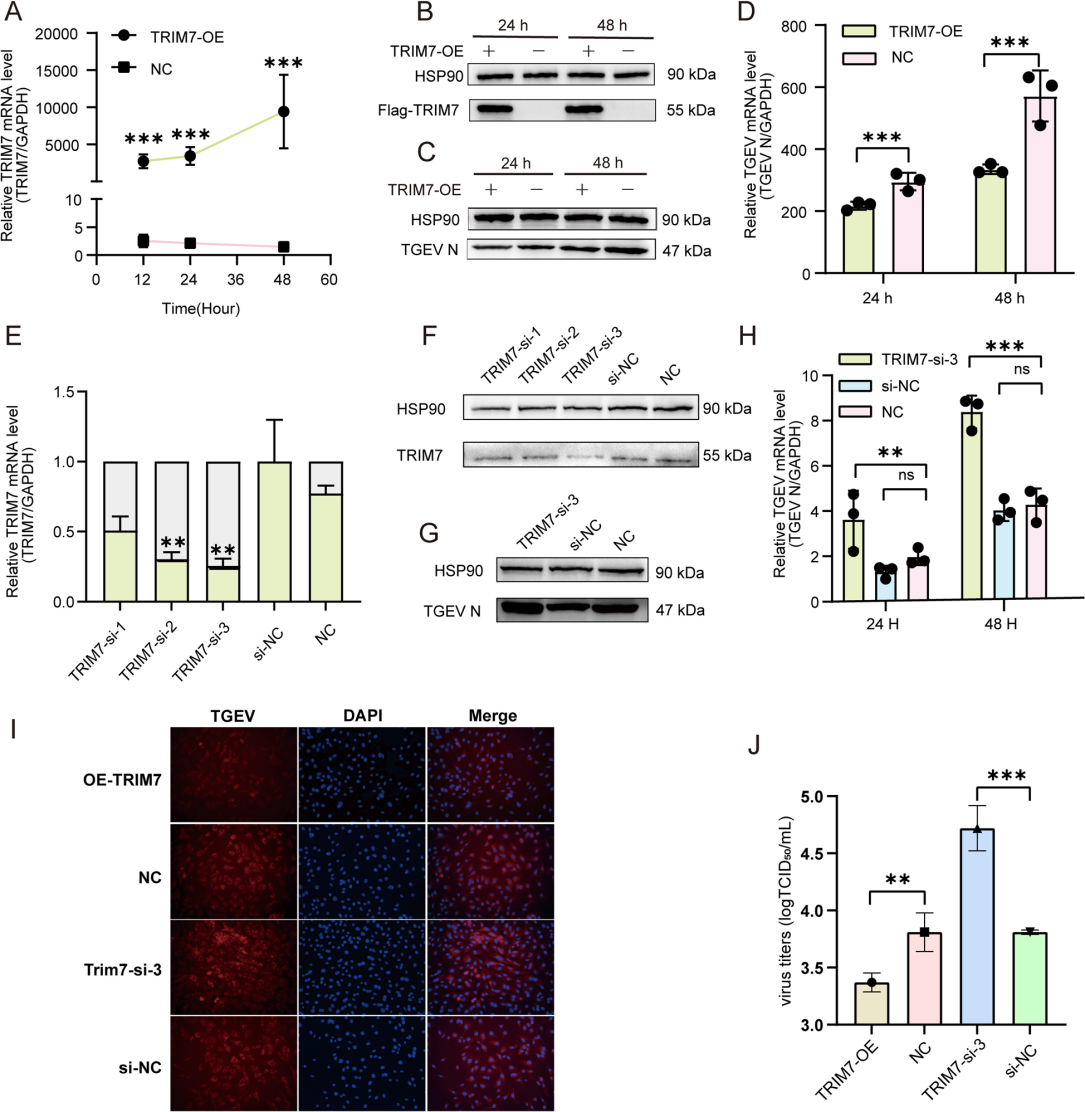
**Functional gain- and loss-of-function experiments confirm TRIM7 as a potential host factor against TGEV. A**, **B** Expression levels of TRIM7 at various time points following the transfection of the TRIM7 overexpression plasmid into IPEC-J2 cells. mRNA levels were measured at 12, 24, and 48 h, with 12 and 24 h showing similar expression levels; therefore, protein detection was performed at 24 and 48 h. **C**, **D** Effects of TRIM7 overexpression on TGEV replication at 24 and 48 hpi. **E**, **F** Efficiency of TRIM7 knockdown in IPEC-J2 cells after 48 h of transfection with siRNAs, si-NC was used as a control. **G**, **H** Impact of TRIM7 interference on TGEV replication. **I** Immunofluorescence assay (IFA) demonstrating the expression and distribution of TGEV in IPEC-J2 cells with TRIM7 overexpression and knockdown. **J** Effect of TRIM7 on TGEV replication assessed by TCID_50_ assay. ns = not significant, **P < 0.01, ***P < 0.001.

To further investigate the role of TRIM7 in TGEV infection, we constructed a TRIM7 overexpression vector and confirmed its efficacy at both the mRNA and protein levels. The results demonstrated that TRIM7 overexpression was successfully achieved (Figures 2A and B). IPEC-J2 cells were subsequently infected with TGEV, and viral replication was assessed at 24 and 48 h post-infection. The results indicated that TRIM7 overexpression significantly inhibited both the mRNA and protein levels of TGEV N (Figures 2C and D). To further validate this, we designed three siRNAs targeting TRIM7, and qRT-PCR and western blot analysis confirmed that TRIM7-si-3 exhibited the highest knockdown efficiency (Figures 2E and F). When TGEV replication was evaluated in TRIM7-si-3-transfected cells, we observed a significant increase in viral replication (Figures2G and H). Consistently, IFA analysis further supported that TRIM7 overexpression inhibited TGEV expression, whereas TRIM7 knockdown promoted viral replication (Figure. 2I). TCID_50_ results showed that TRIM7 overexpression significantly reduced viral titers (*P* < 0.01), while TRIM7 knockdown led to a significant increase in viral titers (*P* < 0.001) (Figure 2J). Collectively, these findings suggest that TRIM7 plays an antiviral role by suppressing TGEV replication.

### TRIM7 binds to the TGEV N protein and promotes its ubiquitination and degradation

**Figure 3:**
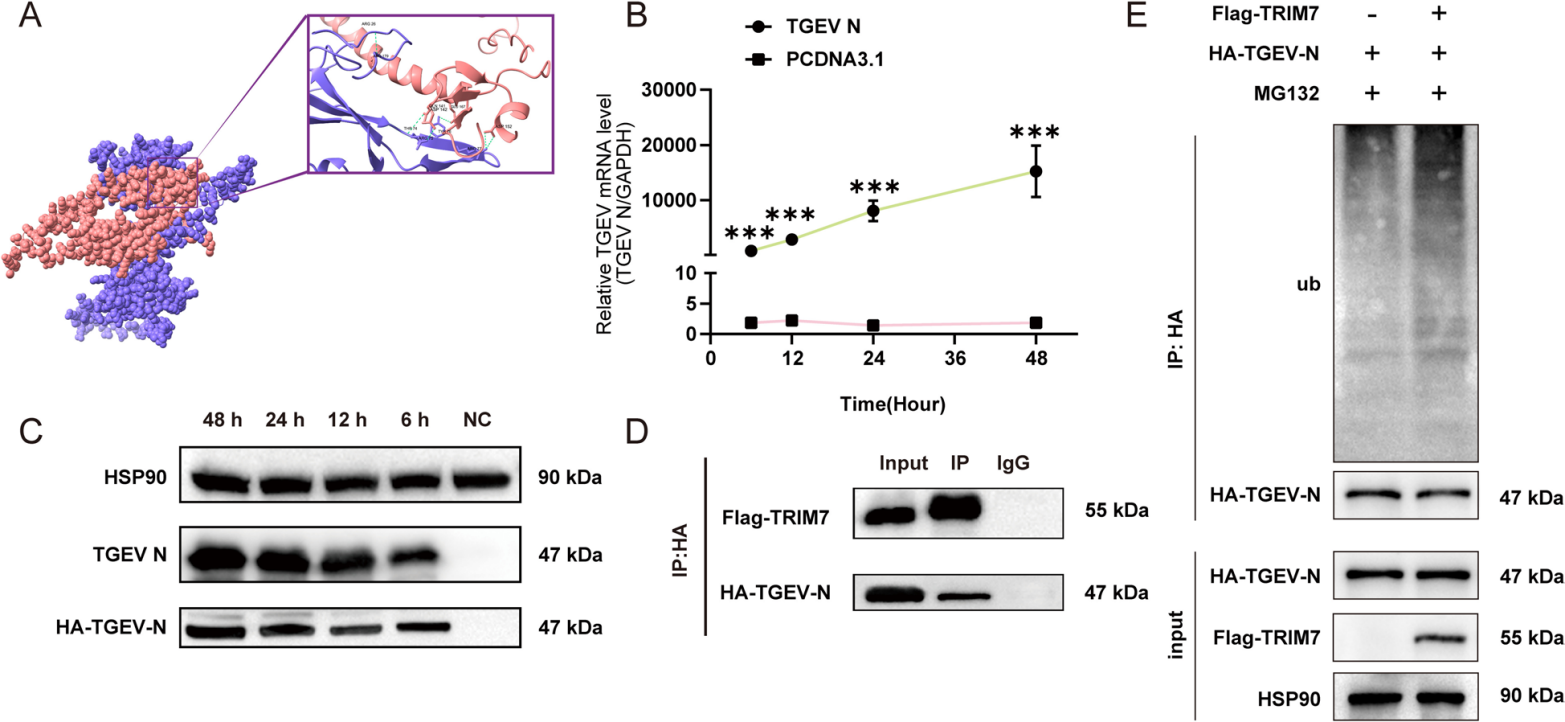
**TRIM7 interacts with the TGEV N protein and regulates its ubiquitination. A** Predicted binding sites and key hydrogen bonds for the interaction between the TGEV N and TRIM7 proteins, with ribbon diagrams colored as follows: TRIM7 (pink) and TGEV N (purple). **B** Detection of TGEV N mRNA expression at different time points following transfection of HA-tagged TGEV N into HEK 293 T cells. The control group was transfected with the empty pCDNA3.1 vector. **C** Western blot analysis of TGEV N and HA-tag expression at different time points following transfection of HA-tagged TGEV N into HEK 293 T cells. **D** Co-immunoprecipitation (Co-IP) assay confirming the interaction between TRIM7 and the TGEV N protein. **E** Co-IP analysis demonstrating the effect of TRIM7 overexpression on the ubiquitination level of the TGEV N protein. Co-IP was performed using HA-tag antibody for pull-down.

Our study demonstrated that the overexpression of TRIM7 suppresses TGEV N protein expression. As an E3 ubiquitin ligase, TRIM7 may facilitate the degradation of the N protein through ubiquitination. To explore this possibility, we employed AlphaFold 3 to predict the interaction between TRIM7 and the N protein, revealing six hydrogen bonds between the two proteins (Figure 3A), which suggests a potential interaction. To validate this, we constructed HA-tagged TGEV N protein and Flag-tagged TRIM7 expression vectors, successfully expressing them in HEK 293 T cells (Figures 3B and C). Co-immunoprecipitation (Co-IP) assays using anti-HA antibody for pull-down confirmed the interaction between TRIM7 and the TGEV N protein, demonstrating that TRIM7 physically associates with the N protein (Figure 3D). Furthermore, after inhibiting protein degradation with MG132, ubiquitination assays demonstrated that TRIM7 overexpression significantly increased the ubiquitination level of the TGEV N protein (Figure 3E), indicating that TRIM7 mediates the degradation of the N protein via ubiquitination.

### TRIM7 overexpression enhances the immune response in IPEC-J2 cells

**Figure 4:**
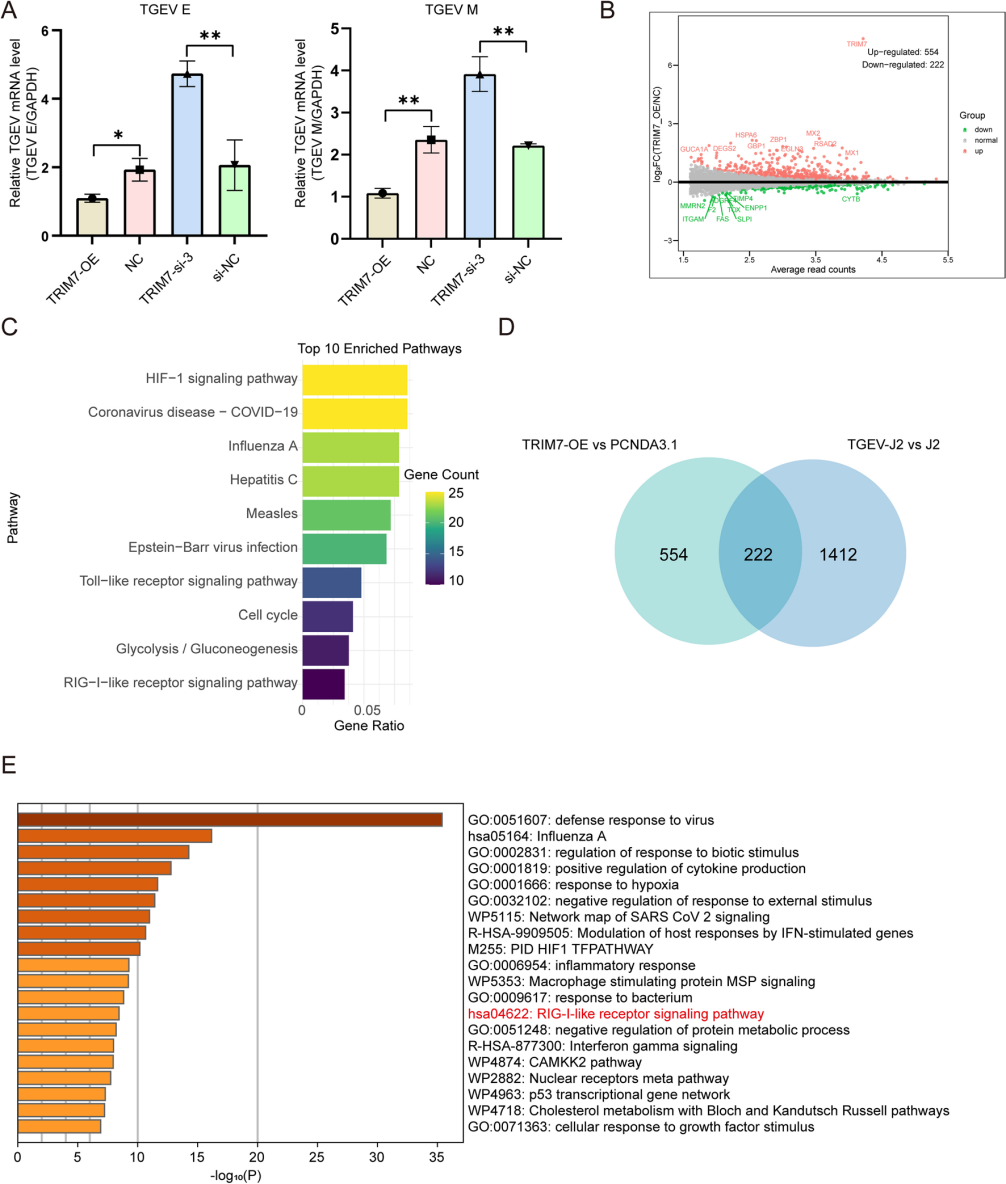
**RNA-seq reveals that TRIM7 overexpression activates host immune responses. A** Effect of TRIM7 on the mRNA levels of TGEV *E* and *M* genes. **B** Volcano plot of differentially expressed genes (DEGs) following TRIM7 overexpression. The x-axis displays the fold change in gene expression (log_2_FoldChange), while the y-axis represents the significance of differential expression (-log_10_ padj). Red dots indicate upregulated genes, green dots indicate downregulated genes, and the blue dashed lines represent the threshold for DEG screening. **C** KEGG enrichment analysis of the upregulated DEGs in TRIM7-overexpressing cells. **D** Integrative Analysis of DEGs from TRIM7 overexpressing RNA-seq data and TGEV infected RNA-seq data, respectively. The left panel illustrates DEGs between the TRIM7 overexpression group and the PCDNA3.1 group, while the right panel depicts the DEGs between the TGEV infection group and the negative control group, and the middle panel displays overlapping genes between the two conditions. **E** Enrichment analysis of overlapping DEGs. Metascape [23] was used for enrichment analysis. ns: not significant, * *P* < 0.05, ** *P* < 0.01, *** P < 0.001.

Using qRT-PCR to assess the mRNA expression of TGEV *E* and *M* genes, we found that TRIM7 overexpression suppressed the expression levels of both *E* and *M*, whereas TRIM7 knockdown led to their upregulation (Figure 4A). These results suggest that TRIM7 may regulate TGEV infection through mechanisms beyond N protein degradation. Therefore, we performed RNA-seq analysis to further explore other potential antiviral pathways mediated by TRIM7. Principal component analysis (PCA) revealed distinct clustering of the samples (Additional file 1A). Differential gene expression (DGE) analysis revealed 554 upregulated and 222 downregulated DEGs in TRIM7-overexpressing cells, among which interferon-stimulated genes like *MX1* and *MX2* were notably upregulated (Figure 4B). These findings suggest that TRIM7 overexpression may trigger an immune response. KEGG pathway enrichment analysis further indicated that the upregulated DEGs were primarily associated with immune response-related pathways, including Coronavirus disease-COVID-19, Toll-like receptor signaling, and RIG-I-like receptor signaling (Figure 4C). Similarly, GO enrichment analysis supported these findings (Additional files 1B and C), indicating an enhanced immune response in TRIM7-overexpressing cells.

A comparative analysis of our RNA-seq data with previously published RNA-seq data from TGEV-infected IPEC-J2 cells [22] identified 222 overlapping genes (Figure 4D). Metascape is an effective and efficient tool for experimental biologists to comprehensively analyze and interpret OMICs-based studies in the big data era [23]. In this study, we used Metascape to analyze the overlapping genes, the results indicated that the RIG-I signaling pathway was also enriched in this dataset (Figure 4E, Additional file 2). Given that previous studies have established a strong link between TRIM family proteins and type I IFN production through the RIG-I signaling pathway [24–28], our findings suggest that the RIG-I signaling pathway may be a key mechanism by which TRIM7 exerts its antiviral activity against TGEV.

### TRIM7 enhances the RIG-I pathway and upregulates downstream genes

**Figure 5:**
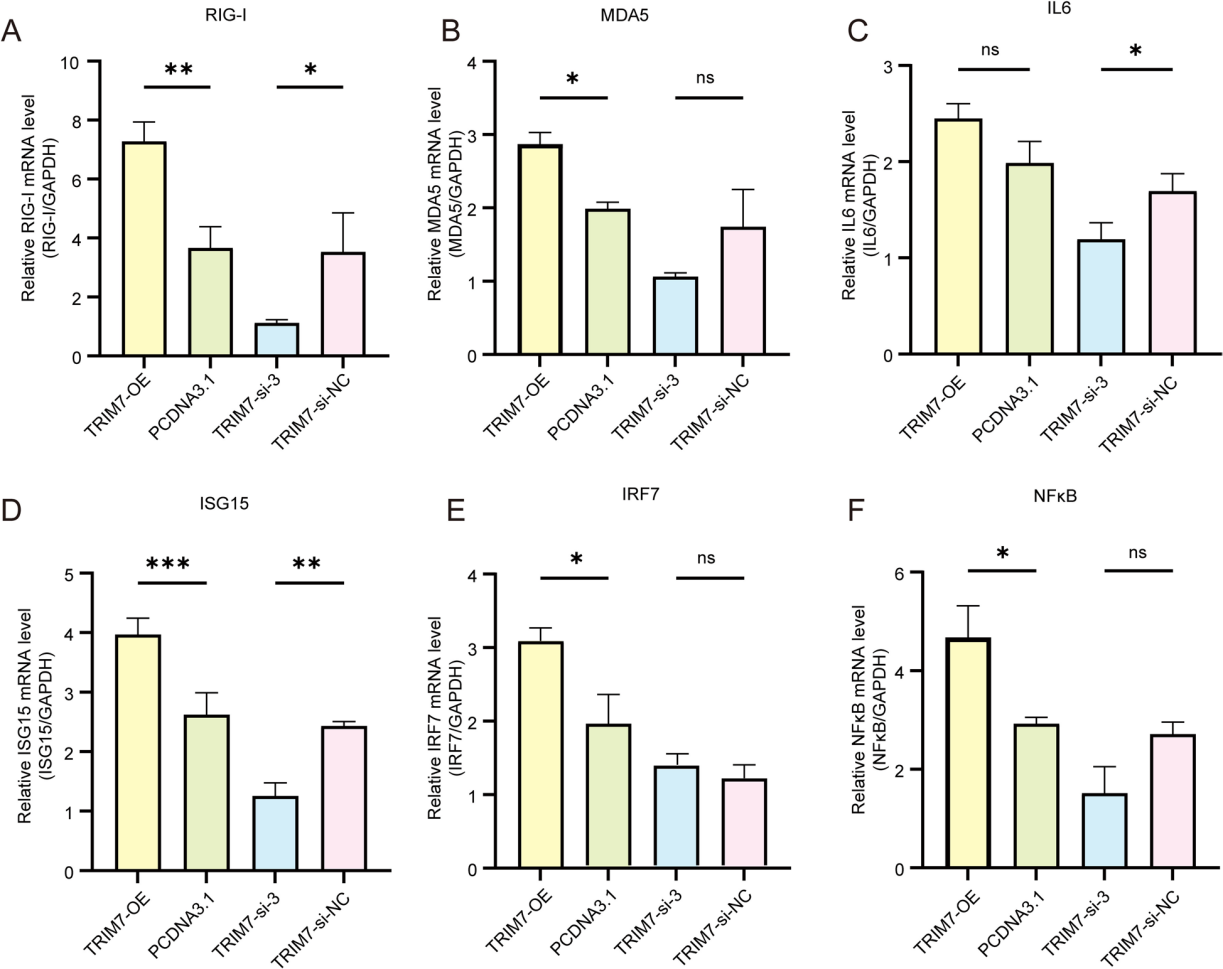
**Effects of TRIM7 overexpression and interference on the RIG-I signaling pathway and downstream gene expression.** ns: not significant, *P < 0.05, **P < 0.01, ***P < 0.001.

The RIG-I signaling pathway is a crucial component of the host defense against viral infections. Our RNA-seq analysis indicated that this pathway may be one of the key mechanisms through which TRIM7 exerts its antiviral effects. To validate this hypothesis, we examined the expression of downstream genes associated with the RIG-I pathway, as well as innate immune factors and inflammatory mediators, in cell lines with TRIM7 overexpression and knockdown. Compared to control groups, TRIM7 overexpression significantly upregulated MDA5, IRF7, and NF-κB (*P* < 0.05), while RIG-I (*P* < 0.01) and ISG15 (*P* < 0.001) exhibited even greater upregulation. Conversely, in TRIM7-knockdown cells, the expression of RIG-I and IL-6 was significantly downregulated (*P* < 0.05), and ISG15 expression was markedly reduced (*P* < 0.01) (Figure 5). These results further support the hypothesis that TRIM7 mediates its antiviral effects against TGEV through the RIG-I signaling pathway.

### TRIM7 activates the RIG-I pathway to induce type I IFN and inhibit TGEV infection

**Figure 6:**
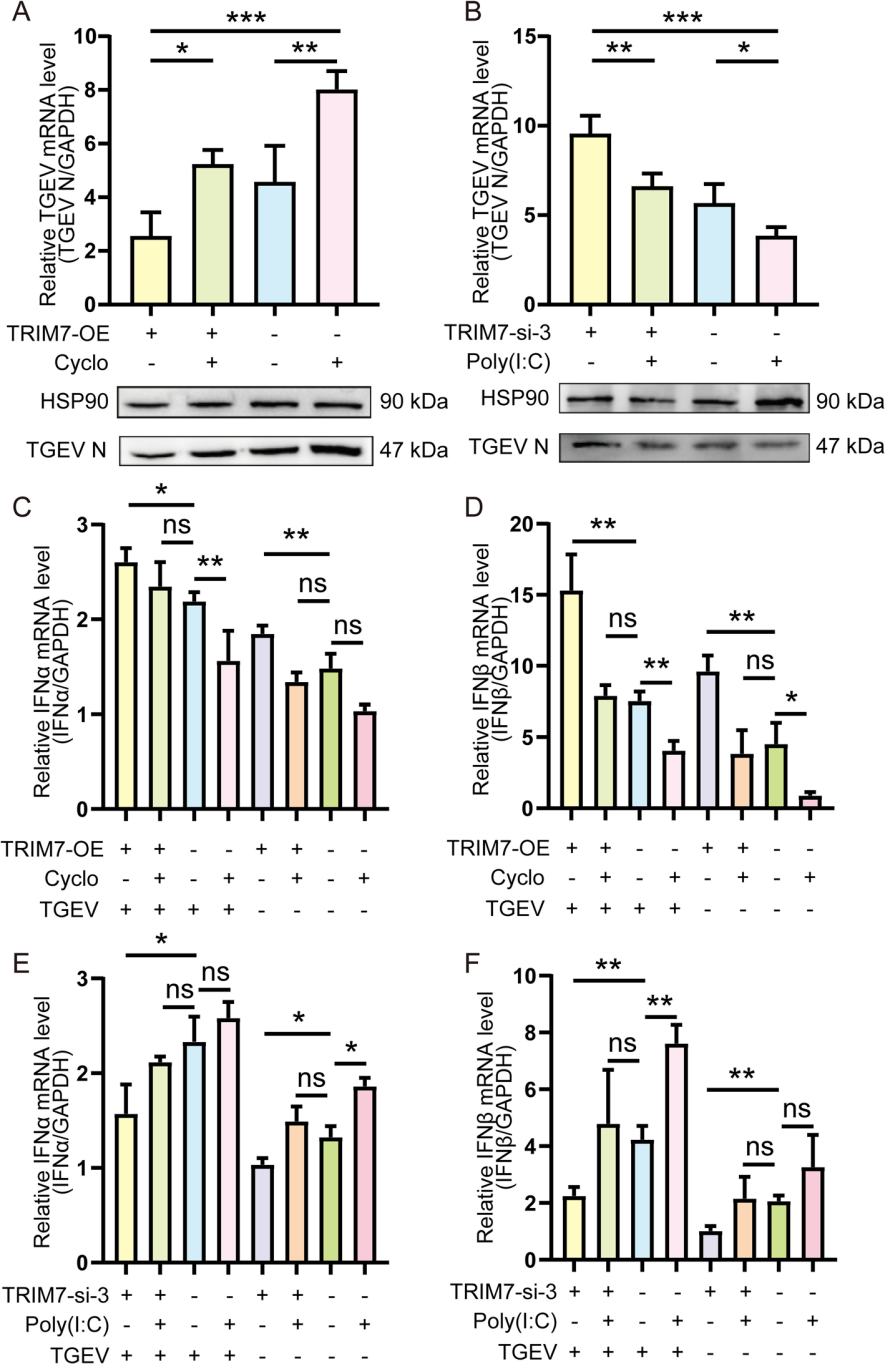
**TRIM7 suppresses TGEV infection by activating the RIG-I pathway. A**, **B** Effects of RIG-I inhibitors and activators on TRIM7-mediated resistance to TGEV Infection. Cyclosporin A (Cyclo) was used to treat TRIM7-overexpressing and control IPEC-J2 cells for 24 h, while Poly(I:C) was administered to TRIM7-interfered and control IPEC-J2 cells for 24 h, followed by TGEV infection for an additional 24 h to assess viral replication. **C**–**F** Effects of RIG-I Inhibitors and Activators on TRIM7-induced type I IFN production. Similar treatments were conducted, and type I IFN levels were measured using qRT-PCR and western blot analysis. ns: not significant, *P < 0.05, **P < 0.01, ***P < 0.001.

To further confirm the role of the RIG-I signaling pathway in TRIM7-mediated antiviral activity, we utilized RIG-I pathway inhibitors and activators. The qRT-PCR and western blot analyses demonstrated that the RIG-I inhibitor Cyclo (MCE, HY-P1934) reversed the inhibition of TGEV replication induced by TRIM7 overexpression. In contrast, the RIG-I activator Poly(I:C) (MCE, HY-107202) counteracted the increase in TGEV replication observed in TRIM7-knockdown cells (Figures 6A and B). Additionally, to determine whether TRIM7 inhibits TGEV by inducing type I IFN through the RIG-I pathway, we examined type I IFN mRNA levels in IPEC-J2 cells. Regardless of TGEV infection status, TRIM7 overexpression effectively induced type I IFN expression, while Cyclo treatment suppressed it. Conversely, TRIM7 knockdown significantly reduced type I IFN expression, whereas Poly(I:C) treatment restored it (Figures 6C–F). These findings suggest that TRIM7 enhances type I IFN production by activating the RIG-I pathway, thereby suppressing TGEV replication in IPEC-J2 cells.

## Discussion

Accumulating evidence indicates that TRIM proteins play a crucial role in host resistance to coronavirus infections [29, 30]. On one hand, TRIMs can induce type I and type II interferons (IFNs), stimulating the production of immunomodulatory cytokines or activating specific immune receptors, thereby modulating viral replication in host cells [17, 31–33]. On the other hand, certain TRIMs directly interact with viral proteins through their C-terminal or SPRY domains, facilitating the targeted degradation of viral structural components [34–38]. As an E3 ubiquitin ligase, TRIM7 contains the conserved RBCC domain characteristic of TRIM proteins, which includes a B-box domain and a C-terminal PRY-SPRY domain. These domains contribute to its functional versatility. Recent studies have underscored the antiviral potential of TRIM7 [14–16], yet its regulatory role in TGEV infection remains unexplored and requires further elucidation.

In this study, we observed that TRIM7 expression was significantly upregulated in IPEC-J2 cells following TGEV infection, suggesting a potential role for TRIM7 in the host's antiviral response. The intestine, as the primary barrier against gastrointestinal viral invasion, rapidly activates early innate immune responses, inducing cytokine production and shaping subsequent adaptive immunity [39]. The high basal expression of TRIM7 in intestinal tissues further supports its involvement in host antiviral defense. Therefore, we investigated the functional role of TRIM7 during TGEV infection. Gain- and loss-of-function analyses demonstrated that TRIM7 is a critical antiviral factor, as its overexpression significantly inhibited TGEV replication, while its depletion led to enhanced viral replication.

Previous studies have demonstrated that TRIM7 can target both structural and non-structural proteins of coronaviruses [40–43]. Furthermore, TRIM7 preferentially interacts with glutamine-terminal motifs in full-length viral proteins, including NSP5 and replication-transcription complexes that contain NSP8 [41, 44, 45]. Based on this information, we hypothesized that TRIM7 may also target viral proteins of TGEV. Through molecular interaction analysis and co-immunoprecipitation (Co-IP), we confirmed that TRIM7 physically interacts with the TGEV N protein and facilitates its degradation via ubiquitination. However, it remains unclear whether TRIM7 exerts additional antiviral effects beyond the degradation of the N protein.

Given that multiple TRIM proteins have been shown to exert antiviral activity through type I and II interferon (IFN) induction, we explored whether TRIM7 exerts similar immunomodulatory effects during TGEV infection. Our findings revealed that TRIM7 overexpression significantly enhanced cellular immune responses. Further pathway enrichment analysis highlighted the RIG-I signaling cascade as a major candidate involved in TRIM7-mediated antiviral regulation. The RIG-I pathway has been extensively associated with the antiviral functions of TRIM family proteins. For example, TRIM25 is crucial for the ubiquitination of RIG-I and the induction of IFN-β [24, 25]. Furthermore, the NS1 of the influenza A virus interacts with TRIM25 to inhibit RIG-I-mediated IFN-β production [26]. Other TRIM proteins, such as TRIM4 and TRIM35, have also been demonstrated to enhance RIG-I-dependent antiviral responses through K63-linked ubiquitination [27, 28]. These findings prompted us to examine whether TRIM7 could similarly regulate RIG-I-mediated signaling to defend against TGEV infection. Interestingly, recent studies have revealed that TRIM7 may also act as a negative regulator of innate immunity under certain conditions. It has been shown to promote K48-linked ubiquitination and proteasomal degradation of MITA/STING, thereby attenuating cytosolic DNA–induced type I IFN responses[46]. In addition, TRIM7 can suppress IFN-β production by inhibiting MDA5-mediated signaling pathways [42]. These studies suggest that TRIM7 may exert virus-specific or context-dependent immune regulatory roles.

In contrast to these findings, our data indicate that TRIM7 enhances antiviral immunity in the context of TGEV infection. This discrepancy suggests that TRIM7 may employ distinct mechanisms depending on the virus or host species involved. In our study, TRIM7 was found to promote RIG-I-mediated IFN-I production in IPEC-J2 cells, thereby suppressing TGEV replication. This hypothesis was further validated using RIG-I activators and inhibitors, confirming that TRIM7 suppresses TGEV replication by activating RIG-I-mediated type I IFN production in IPEC-J2 cells.

**Figure 7:**
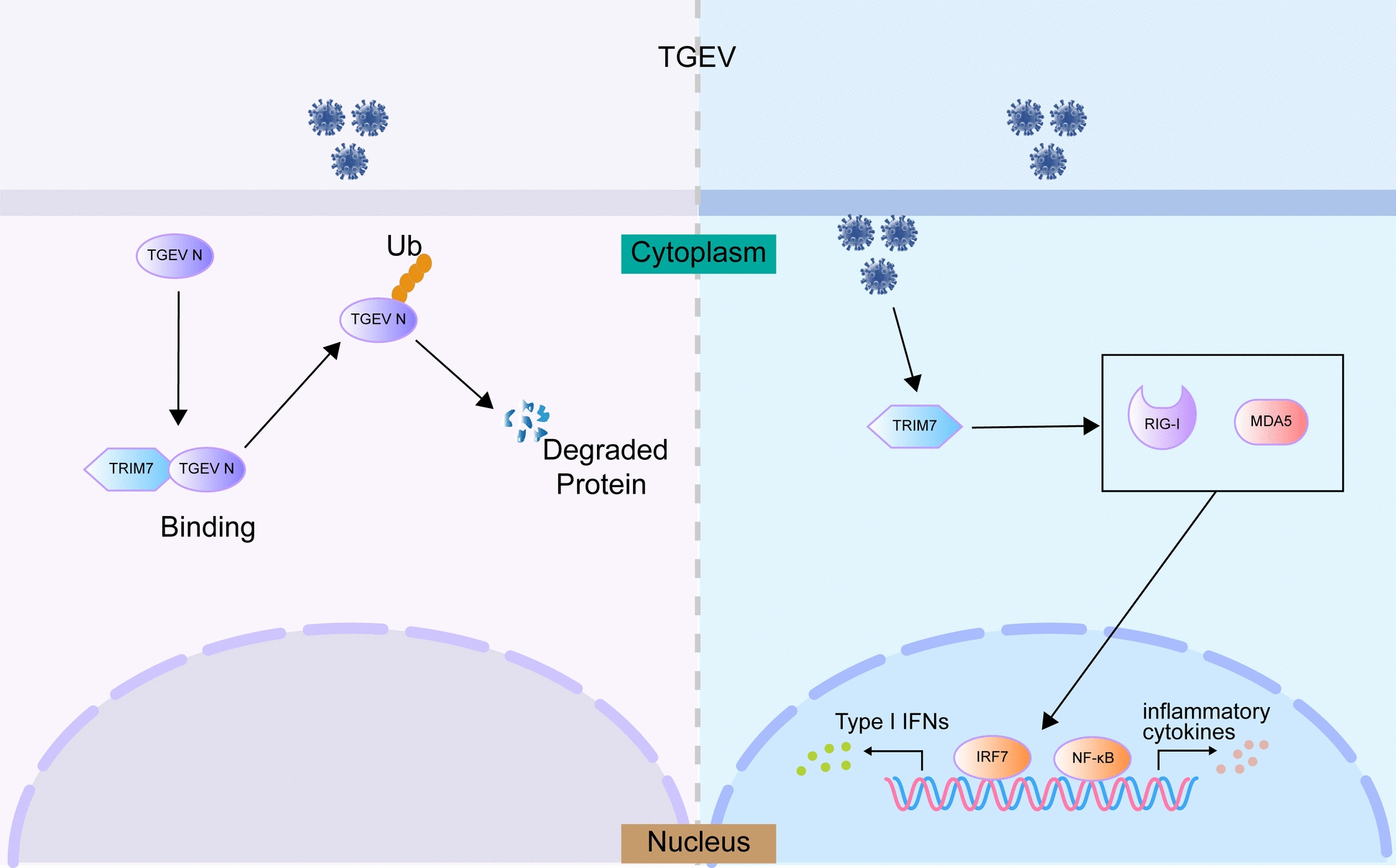
**The antiviral mechanism of TRIM7 against TGEV infection.** On the left, TRIM7 acts as an E3 ubiquitin ligase, catalyzing the ubiquitination of the TGEV N protein. This modification leads to the recognition and subsequent degradation of the N protein via the ubiquitin–proteasome pathway. On the right, TRIM7 activates the RIG-I signaling pathway, which enhances the production of type I interferons and promotes the release of antiviral and anti-inflammatory factors. This dual action significantly contributes to the host's defense against TGEV infection.

Our study provides novel insights into the antiviral role of TRIM7 in combating TGEV infection, emphasizing its dual mechanisms of viral suppression: the ubiquitin-mediated degradation of the N protein and the activation of the RIG-I signaling pathway (Figure 7). These findings indicate that TRIM7 may serve as a promising antiviral target for the control of TGEV and related coronaviruses.

## Supplementary Information

**Additional file 1** **Transcriptome sequencing results**. (A) Principal component analysis (PCA) of TRIM7-overexpressing and control IPEC-J2 cells. (B) GO enrichment analysis histogram. The ordinate is GO term, abscissa is significance level of GO term enrichment, indicated by -log_10_(*padj*), and the *padj* < 0.05 is used as threshold.

**Additional file 2** **Protein-protein interaction network**. We selected a subset of representative terms from the full cluster and converted them into a network layout. More specifically, each term is represented by a circle node, where its size is proportional to the number of input genes fall under that term, and its color represent its cluster identity (i.e., nodes of the same color belong to the same cluster). Terms with a similarity score > 0.3 are linked by an edge (the thickness of the edge represents the similarity score). The network is visualized with Cytoscape with “force-directed” layout and with edge bundled for clarity. One term from each cluster is selected to have its term description shown as label.

## Data Availability

The datasets used and/or analyzed during the current study available from the corresponding author on reasonable request.
