# Supplementary figures and images for: TRIM7 suppresses transmissible gastroenteritis virus replication by targeting the degradation of N protein and activating RIG-I-mediated type I IFN antiviral response
Source: Vet Res. 2025 Nov 5;56:210. doi: 10.1186/s13567-025-01610-z (PMC12587667; doi:10.1186/s13567-025-01610-z)

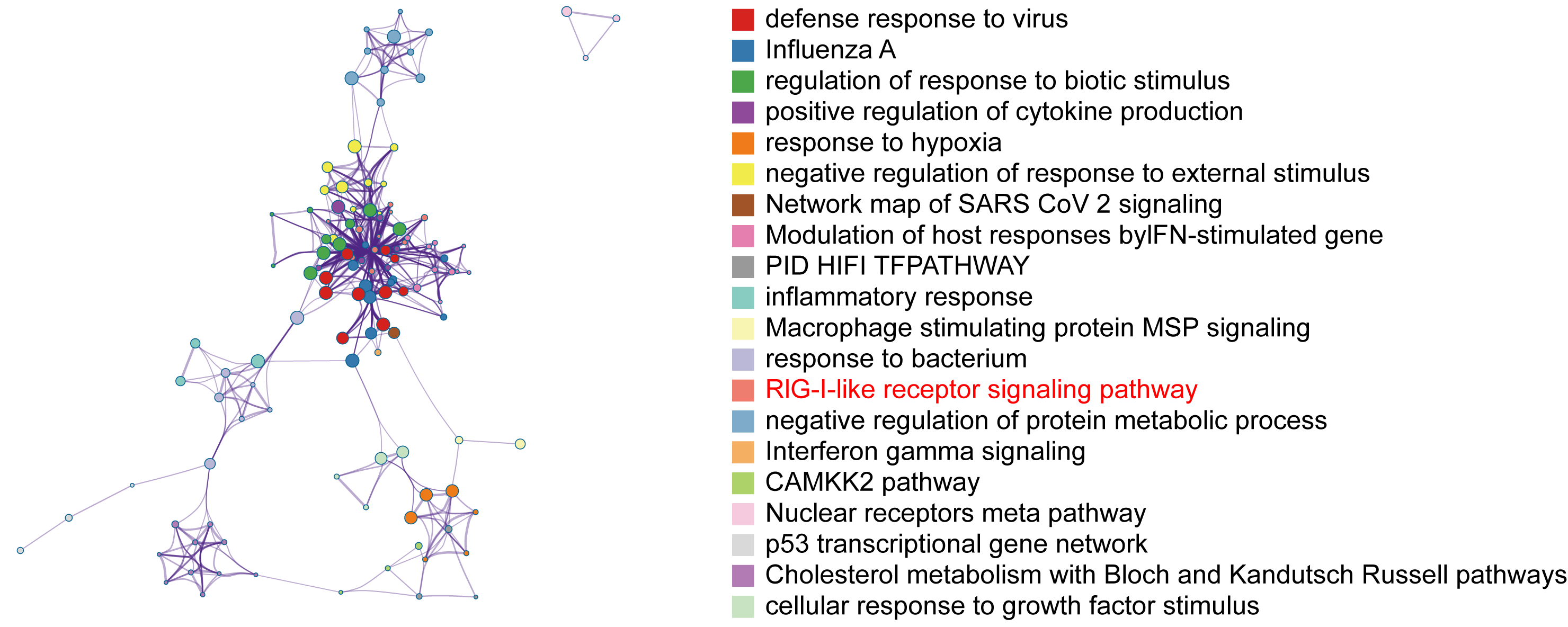

Supplement: Supplementary file 2 — Additional file 2 Protein-protein interaction network. We selected a subset of representative terms from the full cluster and converted them into a network layout. More specifically, each term is represented by a circle node, where its size is proportional to the number of input genes fall under that term, and its color represent its cluster identity (i.e., nodes of the same color belong to the same cluster). Terms with a similarity score > 0.3 are linked by an edge (the thickness of the edge represents the similarity score). The network is visualized with Cytoscape with “force-directed” layout and with edge bundled for clarity. One term from each cluster is selected to have its term description shown as label. [file 13567_2025_1610_MOESM2_ESM.tif]
